# Trial-unique, delayed nonmatching-to-location (TUNL) touchscreen testing for mice: sensitivity to dorsal hippocampal dysfunction

**DOI:** 10.1007/s00213-015-4017-8

**Published:** 2015-07-15

**Authors:** Chi Hun Kim, Carola Romberg, Martha Hvoslef-Eide, Charlotte A. Oomen, Adam C. Mar, Christopher J. Heath, Andrée-Anne Berthiaume, Timothy J. Bussey, Lisa M. Saksida

**Affiliations:** Department of Psychology and MRC/Wellcome Trust Behavioural and Clinical Neuroscience Institute, University of Cambridge, Downing St, Cambridge, CB2 3EB UK; Max-Planck-Institute of Psychiatry, Munich, Germany; Department of Cognitive Neuroscience, Donders Institute for Brain, Cognition and Behaviour, Radboud University Medical Centre, Nijmegen, The Netherlands; Department of Neuroscience and Physiology, New York University Medical Centre, New York, NY USA; Department of Psychology, Concordia University, Montreal, QC Canada

**Keywords:** Mouse, Hippocampus, Delayed nonmatching-to-location, Touchscreen operant chamber, Spatial working memory, Spatial pattern separation

## Abstract

**Rationale:**

The hippocampus is implicated in many of the cognitive impairments observed in conditions such as Alzheimer’s disease (AD) and schizophrenia (SCZ). Often, mice are the species of choice for models of these diseases and the study of the relationship between brain and behaviour more generally. Thus, automated and efficient hippocampal-sensitive cognitive tests for the mouse are important for developing therapeutic targets for these diseases, and understanding brain-behaviour relationships. One promising option is to adapt the touchscreen-based trial-unique nonmatching-to-location (TUNL) task that has been shown to be sensitive to hippocampal dysfunction in the rat.

**Objectives:**

This study aims to adapt the TUNL task for use in mice and to test for hippocampus-dependency of the task.

**Methods:**

TUNL training protocols were altered such that C57BL/6 mice were able to acquire the task. Following acquisition, dysfunction of the dorsal hippocampus (dHp) was induced using a fibre-sparing excitotoxin, and the effects of manipulation of several task parameters were examined.

**Results:**

Mice could acquire the TUNL task using training optimised for the mouse (experiments 1). TUNL was found to be sensitive to dHp dysfunction in the mouse (experiments 2, 3 and 4). In addition, we observed that performance of dHp dysfunction group was somewhat consistently lower when sample locations were presented in the centre of the screen.

**Conclusions:**

This study opens up the possibility of testing both mouse and rat models on this flexible and hippocampus-sensitive touchscreen task.

**Electronic supplementary material:**

The online version of this article (doi:10.1007/s00213-015-4017-8) contains supplementary material, which is available to authorized users.

## Introduction

Decades of studies have demonstrated that the hippocampus is a critical structure for learning and memory (Scoville and Milner 1957; O’Keefe and Dostrovsky 1971; Olton et al. 1979), which is affected in many neurodegenerative and neuropsychiatric disorders including Alzheimer’s disease (AD) and schizophrenia (SCZ) (Small et al. 2011). As a result, many studies of rat and mouse models of these diseases focus on the hippocampus and associated cognitive functions such as memory for locations and spatial navigation (Chishti et al. 2001; Oddo et al. 2003; Pletnikov et al. 2008). Maze-based tests, e.g. Morris water maze or T-maze, and aversive learning tests, e.g. passive avoidance or fear conditioning, are widely used to assess memory and spatial function in mouse disease models (Crawley 2008). Although these tests are popular and well validated for assessing hippocampal function, they have several shortcomings. For example, swimming in cool water or receiving electric shocks is stressors for mice that can potentially confound cognitive results. In addition, the laboratory environment and contact with experimenters are known to affect behavioural outcomes (Crabbe et al. 1999; Sorge et al. 2014). Although there have been efforts to minimise these variables by employing standardised procedures, the results have not been universally successful (Wahlsten 2001; Mandillo et al. 2008).

A touchscreen-based automated operant system for rodent cognition has been developed and used in mice to overcome some of the issues of conventional hand-run tests mentioned above (Bussey et al. 2011; Horner et al. 2013; Mar et al. 2013; Oomen et al. 2013). Advantages of the operant testing system include less aversive task procedures, minimal experimenter contact due to automation and standardisation across laboratories by using the same apparatus and cognitive test programs. Additionally, the use of the touchscreen system enables researchers to run multiple cognitive tests, i.e. a battery approach as is done in humans, within the same test environment. More importantly, cognitive paradigms for mice can be adapted directly from touchscreen-based computerised tests for humans such as CANTAB (Robbins 2006) and vice versa. This similarity could be especially valuable in translating cognitive results between species in preclinical and clinical trials.

The trial-unique nonmatching-to-location (TUNL) task in the touchscreen system allows the assessment of spatial working memory and spatial pattern separation while reducing confounding motor mediating responses (Talpos et al. 2010). The task has previously been demonstrated to be sensitive to hippocampus and prefrontal cortex dysfunction in the rat (Talpos et al. 2010; McAllister et al. 2013), and hence the TUNL task might be valuable to test cognitive deficits related to AD and SCZ. However, many animal models expressing disease-related genetic abnormalities are exclusively available in mice (Papaleo et al. 2012; Webster et al. 2014), and the TUNL task has only been available for the rat. Therefore, in this study, we optimised the TUNL task for use in mice and tested whether it, like the rat version, is sensitive to dorsal hippocampal (dHp) dysfunction.

## Methods and materials

### Subjects

Thirty-two male C57BL/6J mice (Harlan, Bicester, UK) were 8–9 weeks old at the start of the experiment. Throughout all experiments, the same mice were used. Mice were housed between three and four per cage. The holding room maintained a 12-h light cycle (lights off, 7 AM). All experiments were performed during the dark cycle and were in accordance with the UK Animals (Scientific Procedures) Act, 1986.

### Apparatus

The touchscreen automated operant chamber system (Campden Instruments Ltd., UK) was used and details have been described previously (Horner et al. 2013). Briefly, the apparatus is composed of a trapezoidal shaped floor, a touchscreen, a reward delivery magazine (opposite to the screen), two infra-red (IR) beams for motor activity detection (one near the screen and the other near the magazine) and black Perspex sidewalls. Strawberry-flavoured milkshake was provided as a liquid food reward (Yazoo, FrieslandCampina, Ltd., UK). A black Perspex mask with five response windows (each comprised of a 4 × 4 cm square aperture, 1.5 cm above the grid floor) covered the touchscreen to reduce incidental touches. The operant chamber was placed inside a sound- and light-attenuating box with a house light, a tone generator, a ventilating fan and an IR camera. ABET software by Campden Instruments Ltd. was used to control the system and to collect data.

### Behavioural procedures

The training of mice before the TUNL task followed the steps described previously (Horner et al. 2013; Oomen et al. 2013) with a few modifications. In brief, after acclimatisation to the animal facility, food restriction began to maintain 85–90 % of free feeding body weight throughout the experiments. Following habituation to the operant chamber for 20 min, the mice were incrementally trained to touch a white square stimulus presented pseudo-randomly in one of the response windows to receive a reward, and then to initiate the next trial by breaking the IR beam near the reward magazine. Mice were trained until they were able to collect 30 rewards within 45 min. For the last step of pretraining, a touch made to blank windows was followed by a 5-s time-out signalled by illumination of the house light. The same trials were repeated (correction trials) after a 5-s inter-trial interval (ITI) until the mouse made a correct response, but these correction trials were not counted when calculating performance. Reward collection initiated a 15-s ITI for the next trial. After completing 48 trials within 30 min at over 80 % correct for two consecutive sessions, the mice were moved onto TUNL training.

**Fig. 1:**
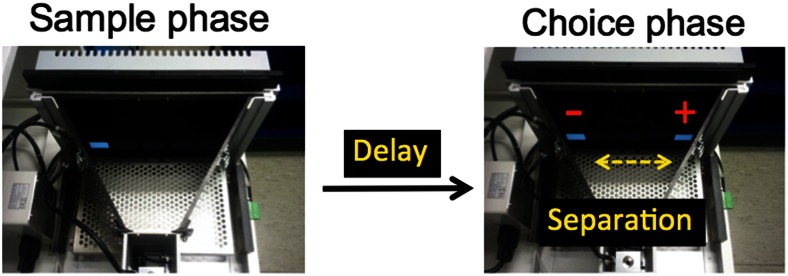
The TUNL task. The screen is covered with a black Perspex mask with five response windows. In the choice phase, the correct stimulus (white square) denoted by “+” is in a new location that does not match the sample location denoted by “−”. This trial has a spatial separation level of 3 (S3), i.e. number of response windows between the two stimuli is 3 (see Table 1)

In all experiments, a trial of the TUNL task was composed of two phases (see Fig. 1). In the sample phase, the initiation started one stimulus presentation (a white square) in one of the five possible locations on the screen. The initiation was done by breaking the IR beam near the reward magazine which was 3 cm from the magazine, not the IR beam in the magazine. This was to reduce the travel time between the screen and the initiation area. Following a nose-poke to this stimulus (the *sample*), the stimulus disappeared. After a delay, a second initiation procedure (either by an IR beam break near the magazine in experiments 1–3 or a head entry to the magazine in experiment 4) was required for the start of next choice phase. The second initiation was designed to prevent mice from mediating during the delay period by waiting in front of to-be-correct or to-be-incorrect location for the next choice phase. In the choice phase, two stimuli were presented: one in the old (sample, incorrect) location, the other in the new (correct) location. A touch to the correct location resulted in delivery of a reward and an ITI for the next trial, but a touch to the incorrect location resulted in a 5-s time-out and then an ITI, either followed by correction trials in experiments 1–3 or by the next new trial in experiment 4. Correction trials followed the same procedures as normal trials, except that the same sample and choice locations from the previous incorrect trial continued until the correct choice was made.

Experiment 1 was composed of stages 1 and 2 TUNL training (see Table 1). For stage 1, training was conducted by presenting the sample stimulus in non-centre locations. First, the two corner locations were used as the sample and choice locations, which is the maximum spatial separation level (S3) possible for two stimuli in the choice phase (20 cm apart). Once an individual mouse reached the criterion, i.e. average of 70 % correct over two sessions, the level of separation was reduced to S2, then to S1. Task parameters were as follows: 2 s delay, 15 s ITI, 5 s correction trial ITI, 5 s time-out, a reward for one in three sample touches, initiation was required for both sample and choice phases by breaking the IR beam near the reward magazine, and the session finished after 45 min or completing 36 trials, whichever came first.

**Table 1:**
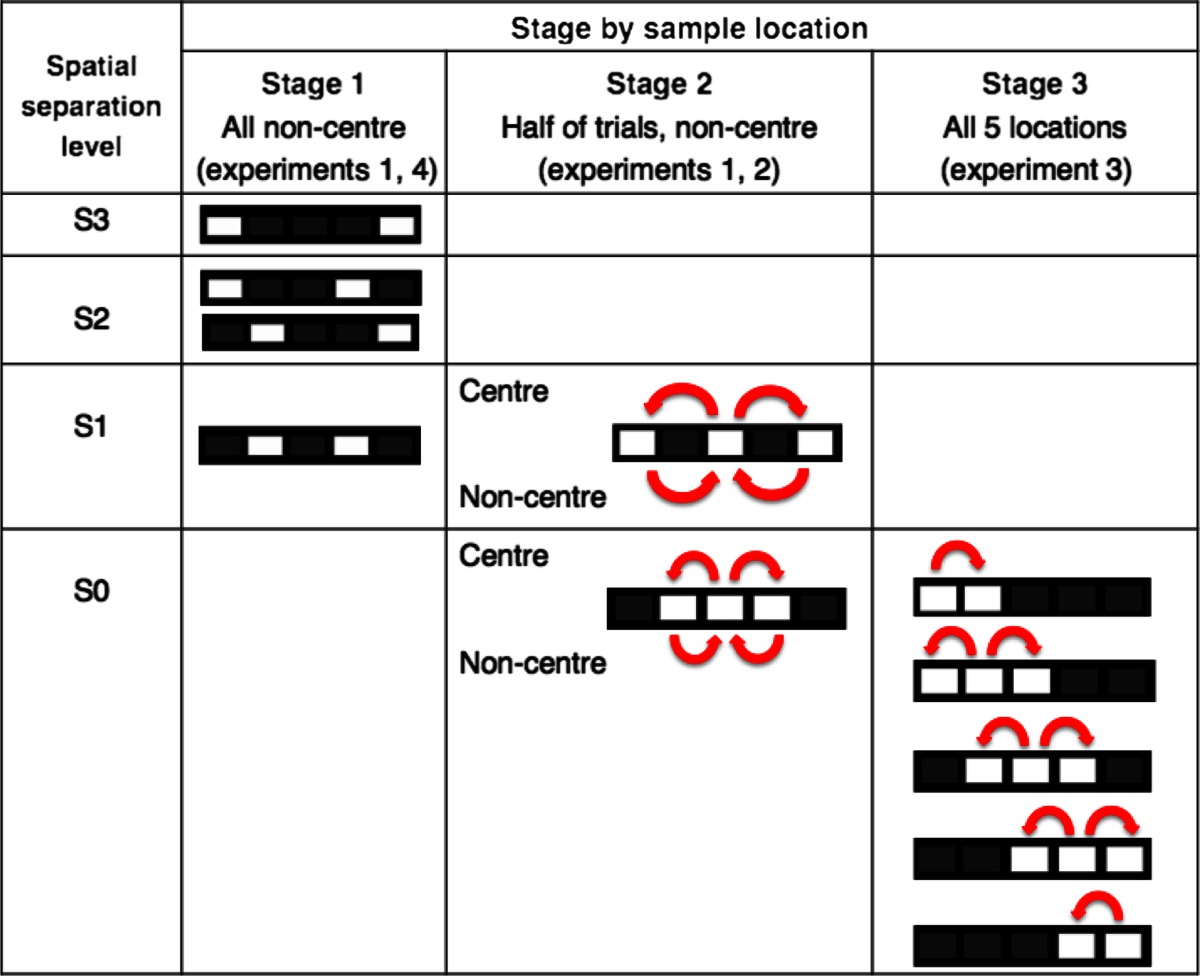
Training steps by sample location and spatial separation level. For stage 1, white squares represent a pair of stimuli presented in the choice phase within five possible locations at each separation level. For stages 2 and 3, arrows indicate possible pairs of locations used during a given trial; the start of an arrow indicates a sample location and the end of the arrow indicates the correct location within that pair

For stage 2, the centre location was also used as a sample location. It was important that all locations were rewarded equally often, so the number of sample (incorrect) and choice (correct) stimulus presentations for each location in the choice phase was matched. To ensure this, the sample stimulus was presented in the centre in half of the trials and in the corners in the other half (see Table 1, stage 2). Otherwise, mice may develop a preference for the more frequently rewarded locations. For example, when sessions with only sample centre trials were used, we observed that mice rapidly developed a preference for non-centre locations in the choice phase (data not presented). All mice were trained on separation S1 until group performance became stable, and then were trained on S0. To see the effects of separation level, S0 and S1 conditions were mixed within a session until performance stabilised. Then the average performance of the last two sessions was used for analysis. Finally, to examine the effects of delay on performance, the delay between sample and choice phases was varied between sessions. Mice were exposed to two consecutive sessions of either 0, 3 or 6 s delay with the order of exposure randomised but balanced across the cohort such that equal numbers of mice received the same delay on a given day. The average performance for each delay was used for analysis. Task parameters during stage 2 were the same as those in stage 1 with the exception of baseline training at a 0-s delay, no reward for sample touches and the session finishing after 60 min or completion of 48 trials, whichever came first.

In experiment 2, the same cohort of mice from experiment 1 was allocated into either excitotoxic dHp lesion or sham surgeries. After recovery from surgery, performance on the TUNL task was re-established in stage 2 (S1, 0-s delay). Following this, effects of delay were tested at a fixed separation S1. Two mixed-delay conditions were run until the performance became stable: first mixed 0 and 2 s sessions, then mixed 0 and 4 s sessions. Next, to test the effect of separation level, mixed S0 and S1 sessions were run at a fixed 0-s delay. Average performance of the last four sessions was used to analyse delay and separation effects.

In experiment 3, based on the results from experiment 2, we hypothesised that the degree to which animals can predict the correct location in the choice phase might affect TUNL performance. That is when sample location is in the centre, the correct location can be either to the right or to the left side of the sample location (low predictability), but when sample location is off-centre, the correct location is more likely to be to the left if the sample is to the right of centre, or to the right if the sample is to the left of centre (high predictability). To investigate this idea, in experiment 3 we ran a task condition that maximised the number of low predictability trials. To maximise the number of low predictability trials, all five locations with a separation of S0 was used with number of rewards equilibrated for each location (stage 3 in Table 1). After performance stabilised at 0 s delay, the delay was increased to 4 s. The last three sessions of each delay were averaged for analysis.

In experiment 4, following experiment 3, we studied the effects of delay and separation in highly predictable conditions, i.e. stage 1. However, sessions containing only sample non-centre trials, which have high predictability as described above, are prone to motor mediation behaviours— such as waiting in front of to-be-correct or to-be-incorrect location or positioning the body in certain postures—to bridge the delay without using spatial working memory (Chudasama and Muir 1997). To minimise such confounds, ¼ of full rewards (5 μl) were delivered in a temporally unpredictable way during the delay. Specifically, for every 3-s period during the delay, a ¼ reward was delivered at a randomly selected epoch within that 3-s period. Also, mice were required to exit the reward magazine to initiate the next ¼ reward. By using these procedures, all mice repeatedly entered and exited the reward magazine area during the delay. No visible mediating behaviours were observed. Due to the longer delays used in experiment 4, ITI was increased from 15 to 30 s to make the ITI period distinctive from the delay. This also led to removal of correction trials in order to have more trials within the 60-min session limit. First, sessions of trials with mixed 0 and 6 s delays at a fixed separation of S3 were run until performance stabilised. Then, two conditions of mixed-separation levels were tested at a fixed 0-s delay: mixed S3 and S2 sessions, and mixed S3 and S1 sessions. Lastly, to test the effects of longer delays, we returned to mixed-delay conditions: mixed 0, 6 and 9 s sessions and a mixed 0, 9 and 18 s sessions. Statistical analyses were done using the average of the last two sessions in each condition.

### Surgery

Mice were kept under anaesthesia with isoflurane gas and mounted in a stereotaxic frame (David Kopf Instruments, Tujunga, CA, USA). Intraperitoneal (IP) meloxicam (Metacam, Boehringer Ingelheim, Bracknell, UK; 1 mg/10 ml in PBS, 0.1 ml per 10 g body weight) was given for perioperative pain control. The dorsal skull was exposed and the frame was adjusted to align a horizontal plane of the frame with an imaginary line connecting lambda and bregma. For the hippocampal lesion group, holes were drilled and injections of 10 mg/ml NMDA (Sigma, UK) in phosphate-buffered saline (PBS) solution were given at the four coordinates: anteriorposterior (AP), −1.7; lateral (L), ±1.0; ventral (V), −1.9 and AP, −2.3; L, ±1.7; V, −1.9 (in mm, AP and L from bregma, V from the surface of the skull) at a volume of 0.1 and 0.2 μl, respectively. The injections were made using a 5-μl Hamilton syringe fitted with 33 gauge needle at a rate of 0.1 μl/min. After each injection, the needle was left in situ for 4 min before being fully withdrawn. For the sham group, holes were made at the same coordinates and the same needle without NMDA was lowered through the cortex (−1.0 mm from the surface of the skull) but not inside the hippocampus. This was done to match the potential cortical damage by the needle in both lesion and sham groups. After suturing the scalp, the mouse was observed in a recovery chamber (30 °C) until it became mobile. Mice were individually housed overnight, then rehoused to their original cages. Diazepam IP injections (10 mg/10 ml in ethanol and PBS, 0.1 ml per 10 g) were given when seizures were observed. For at least 2 weeks, the mice were given unlimited food until they had regained stable weights. This was also to give enough time for the excitotoxic lesion to develop. Prior to testing, food restriction started again to maintain 85–90 % of free feeding body weight, which typically took 5 to 7 days.

### Data analysis

All data were checked for normality by the Shapiro-Wilk test and for homogeneity of variance by Levene’s test before further analysis. Repeated measures ANOVA (rmANOVA) were performed with appropriate within- and/or between-subject factors. Violation of sphericity assessed by Mauchly’s test was corrected by the Greenhouse-Geisser method. When an interaction was found, simple main effects analyses were conducted for each factor with the Sidak correction. All statistical analyses were conducted using SPSS version 22.

## Results

### Experiment 1: TUNL training for mouse (stages 1 and 2)

In several pilot studies, we have found that mice could not acquire the TUNL task using the same task parameters to those used for training rats (Romberg, unpublished), i.e. using many locations arranged in rows as well as columns, and sessions in which all spatial separations (between sample and correct location) were mixed pseudo-randomly within a session. Therefore, we began by training using two locations (see Table 1, stage 1), with a view toward moving to a more complete, more trial-unique version of the task using more locations and separations as is routinely used in the rat (see Table 1, stage 2).

**Fig. 2:**
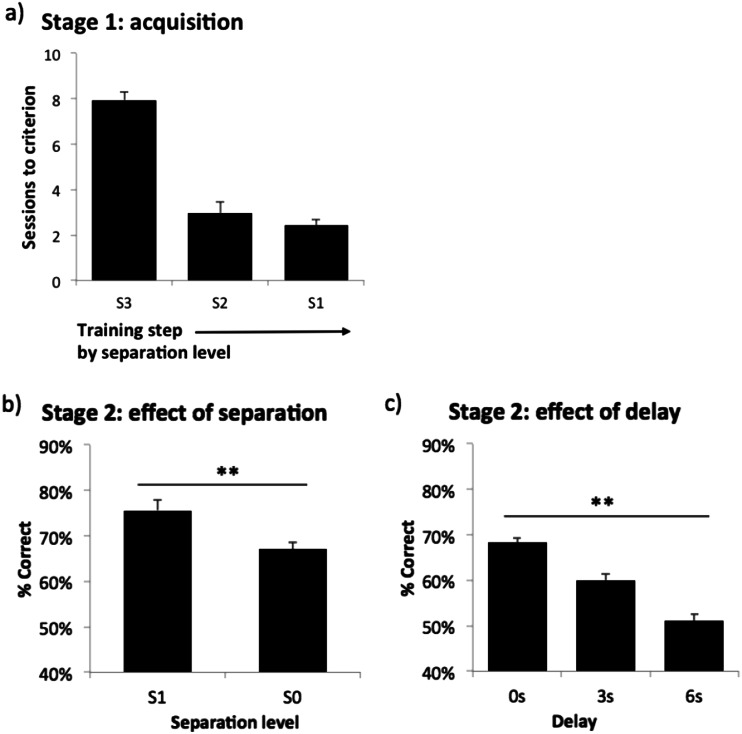
Rate of acquisition of mouse TUNL training, and sensitivity of performance to separation and delay. **a** Number of sessions taken to reach the criterion for each spatial separation level in stage 1; **b** effect of separation in stage 2 under mixed S1 and S0 condition (0 s delay); **c** effect of delay in stage 2 (mixed S1 and S0). Data are presented as mean ± standard error of the mean (SEM). **p < .005

All the mice were able to acquire the stage 1 training steps from separation level S3 to S1 (see Fig. 2a). The mean total number of sessions necessary for stage 1 was 13.3, SD 0.68, min 6, max 24. The training continued on to stage 2 until performance became stable. Then effects of separation level and delay were separately assessed as described in the “Methods and materials” section. There were main effects of separation level (*F*(1,31) = 26.10, *p* < .001: see Fig. 2b), in which performance was lower when the separation was smaller, and delay (*F*(2,62) = 46.95, *p* < .001: see Fig. 2c), in which performance was lower when the delay was longer. In summary, the mice were able to acquire the modified version of the TUNL task within a reasonable amount of time. Task performance was dependent on both separation level and delay. Thus, this experiment indicated that, with appropriate modifications, mice are capable of learning a multiple-location version of TUNL.

### Experiment 2: effects of hippocampal dysfunction on mouse TUNL (stage 2)

After establishing a multiple-location TUNL protocol suitable for mice, we investigated whether damage to the hippocampus in the mouse would have the same hippocampus-sensitivity as seen previously with rats (Talpos et al. 2010).

#### Histology

**Fig. 3:**
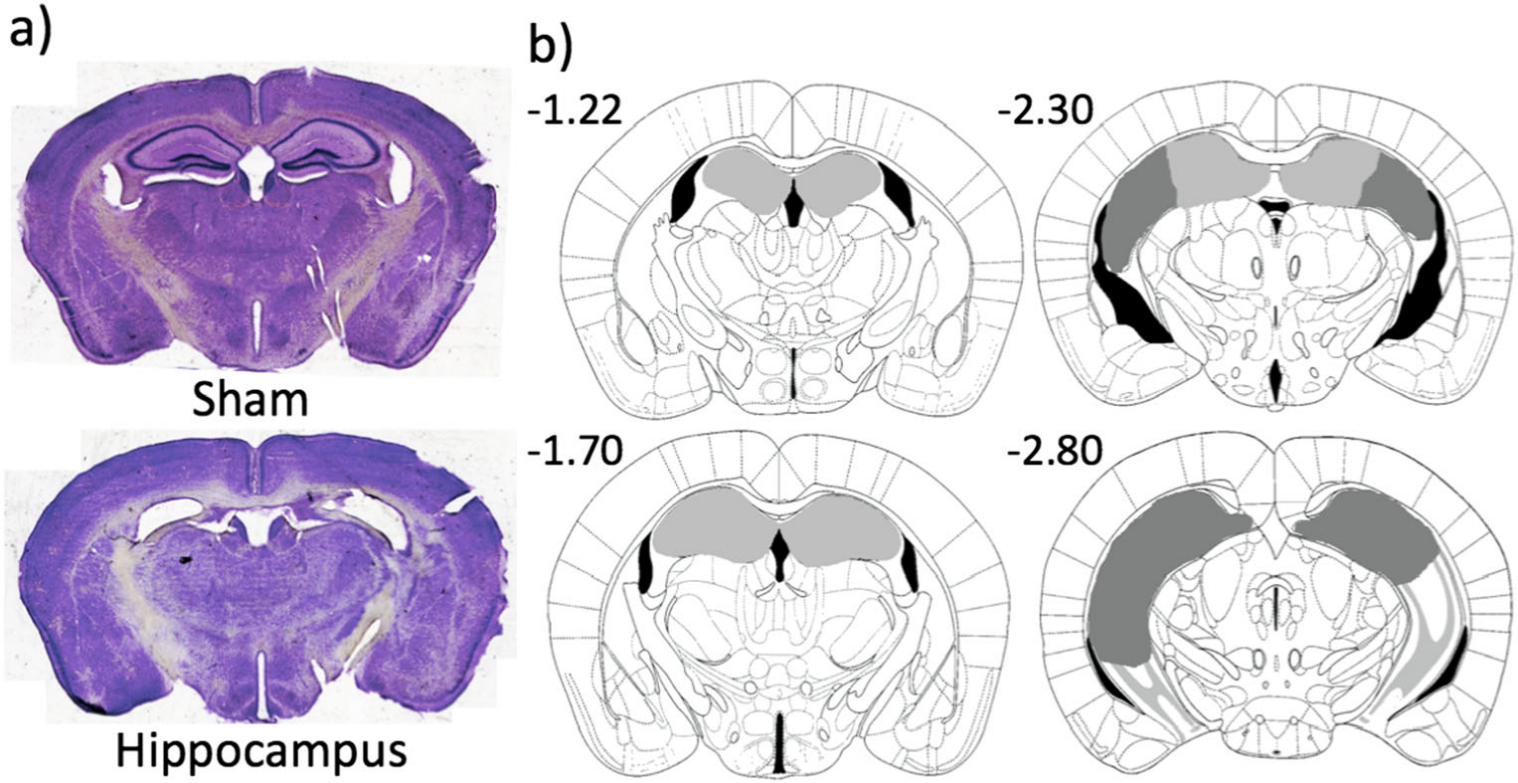
Photographs of representative sections and diagram of the extent of dorsal hippocampal lesions. **a** Photographs of coronal sections corresponding to −1.7 mm from bregma; **b** *light grey* represents the smallest lesion and *dark grey* represents the largest. All numbers correspond to distance in millimetre from bregma

Two mice died during or recovering from surgery: one from the lesion and the other from the sham group. All 15 mice in the lesion group had complete bilateral dorsal hippocampal damage, which was defined anterior to −1.7 mm from bregma (see Fig. 3). In 4 out of 15 mice, there was bilateral ventral hippocampal damage, which was posterior to −2.8 mm from bregma. But in all four cases, more than half of the ventral hippocampus was spared. Bilateral cortical damage was seen in six mice, but there was no significant difference in performance when compared with a non-bilateral cortical damage group (average performance of the last three sessions of post surgery re-acquisition; *t*(13) = .91, *p* = .380).

#### Post surgery re-acquisition of TUNL

**Fig. 4:**
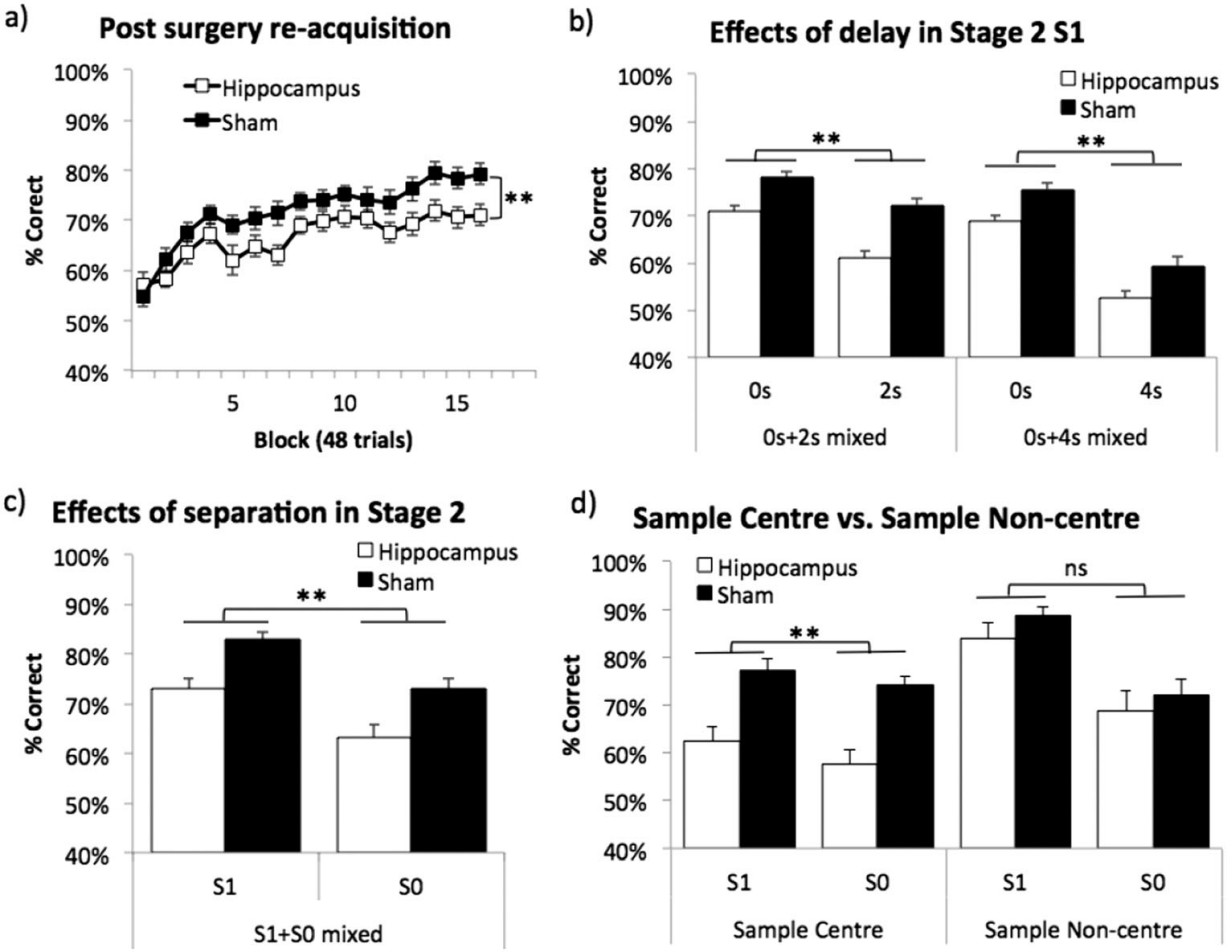
Effects of hippocampal lesions on TUNL. **a** Post surgery re-acquisition in stage 2 (S1, 0 s delay); **b** effects of delay in two mixed-delay conditions in stage 2 (S1); **c** effects of spatial separation in a mixed S1 and S0 condition in stage 2 (0 s delay); **d** detailed analysis of **c** by sample location. Data are presented as mean ± standard error of the mean (SEM). *ns* denotes not significant, **p < .005 main effect of lesion

One month following surgery, although retention of the task was poor, both groups were able to re-acquire the task over training (main effect of block: *F*(15,420) = 16.11, *p* < .001; see Fig. 4a). Re-acquisition of the task in stage 2 (S1, 0 s delay) was worse in the hippocampal lesion group than the sham group (main effect of lesion: *F*(1,28) = 11.69, *p* = .002) and performance on sample centre trials was lower than that on sample non-centre trials (main effect of sample location: *F*(1,28) = 47.9, *p* < .001; data not shown). However, there was no interaction between any factors.

Mixed-delay sessions (see Fig. 4b) revealed main effects of lesion (*F*(1,28) = 33.97, *p* < .001), delay (*F*(1,28) = 46.37, *p* < .001) and sample location (*F*(1,28) = 21.75, *p* < .001) in the mixed 0 and 2 s condition. The same was true for the mixed 0 and 4 s condition (lesion: *F*(1,28) = 13.42, *p* = .001; delay: *F*(1,28) = 178.39, *p* < .001; sample location: *F*(1,28) = 5.26, *p* = .029). Also, a delay by sample location interaction was found in both delay conditions (0 and 2 s mixed: *F*(1,28) = 14.89, *p* = .001; 0 and 4 s mixed: *F*(1,28) = 112.42, *p* < .001, data not shown). Simple main effects analysis showed that the effect of delay was only in the sample non-centre trials but not in the sample centre trials (*F*(1,28) = 37.19, *p* < .001; *F*(1,28) < 1, *ns*, respectively). No other interaction between lesion and other factors was found.

Sessions in which separation level was varied (see Fig. 4c, d) revealed main effects of lesion (*F*(1,28) = 64.12, *p* = .001), separation level (*F*(1,28) = 41.42, *p* < .001) and sample location (*F*(1,28) = 16.05, *p* < .001). In addition, a significant lesion by sample location interaction was shown (*F*(1,28) = 4.89, *p* = .035) and further simple main effects analysis indicated that the hippocampal lesion effect was only in the sample centre trials but not in the sample non-centre trials (see Fig. 4d: *F*(1,28) = 24.72, *p* < .001; *F*(1,28) < 1, ns). No other interactions were found.

#### Motor activity and latencies

**Fig. 5:**
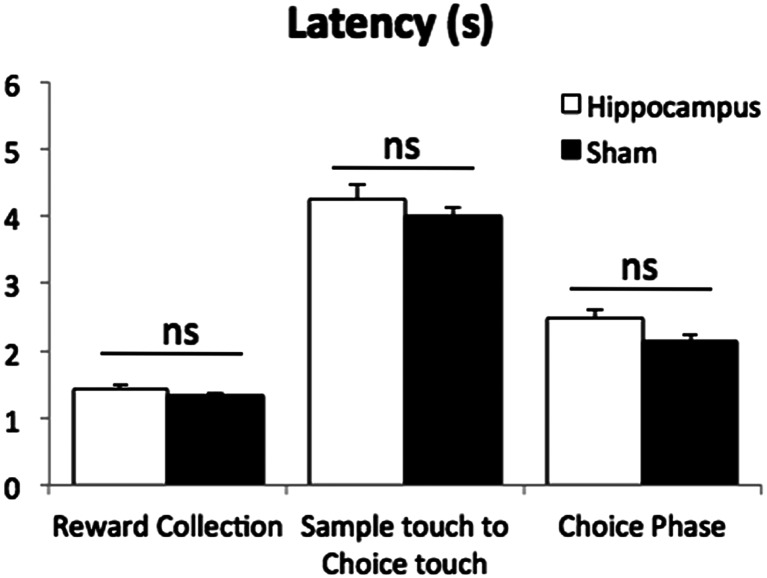
Mean latencies during the post surgery re-acquisition without programmed delay (0 s delay). The “Sample touch to Choice touch” data in the middle indicates there was an approximately 4 s latency between the sample and choice responses under the 0-s programmed delay condition. This was due to travel time between initiation and screen response. Data are presented as mean ± standard error of the mean (SEM). *ns* denotes not significant

Motor activity was measured by the number of IR beam breaks (near the reward magazine) per minute using the average of the last three sessions during the post surgery re-acquisition. The hippocampal lesion group, *M* = 9.56, SD = 2.33, showed significantly higher number of beam breaks compared to the sham group, *M* = 7.05, SD = 1.56 (*t*(28) = 3.46, *p* = .002). Using the same sessions, latencies were calculated as median latency per session, rather than mean, to minimise the effects of anomalously high values within a session. There were no significant differences in any of latency measures, which included reward collection latency (see Fig. 5; *t*(28) = 0.23, *p* = .225), sample touch to choice touch latency (*t*(23.84) = 1.01, *p* = .323) and choice phase latency (*t*(22.98) = 1.96, *p* = .063). An approximately 4 s latency between sample touch and choice touch (see Fig. 5 middle) means there was an *actual* delay between sample and choice responses caused by the mouse moving between the screen and the IR beam near the reward magazine for initiations, even in the no programmed delay (0 s delay) condition. Thus, 0 s refers to the programmed, minimum delay, rather than the actual delay.

To summarise, the task was very sensitive to dHp lesions. However, in contrast to what has been previously observed in the rat (Talpos et al. 2010), there were neither lesion by delay nor lesion by separation level interactions. This is likely due to the fact that in the current experiments the hippocampal lesion effect was significant even in the baseline condition, e.g. 0 s or S1 condition. By analysing the trials by sample locations, we found that in one case (the mixed-separation sessions; Fig. 4c, d), sample location interacted with the lesion, i.e. hippocampus-lesioned mice were impaired on sample centre trials only. We next conducted further experiments to further explore this sample location effect.

### Experiment 3: sample all locations (stage 3)

In experiment 2 with mixed-separation sessions (see Fig. 4c, d), hippocampus-lesioned mice were impaired on sample centre trials only. We hypothesised that this may have been because of potential differences between the two trial types in the degree to which animals can predict the correct location: when sample location is in the centre, the correct location can be either to the right or to the left side of the sample location (low predictability), but when sample location is off-centre, the correct location is more likely to be to the left if the sample is to the right of centre or to the right if the sample is to the left of centre (high predictability). To investigate this idea, in experiment 3, we ran a task condition that maximised the number of low predictability trials.

**Fig. 6:**
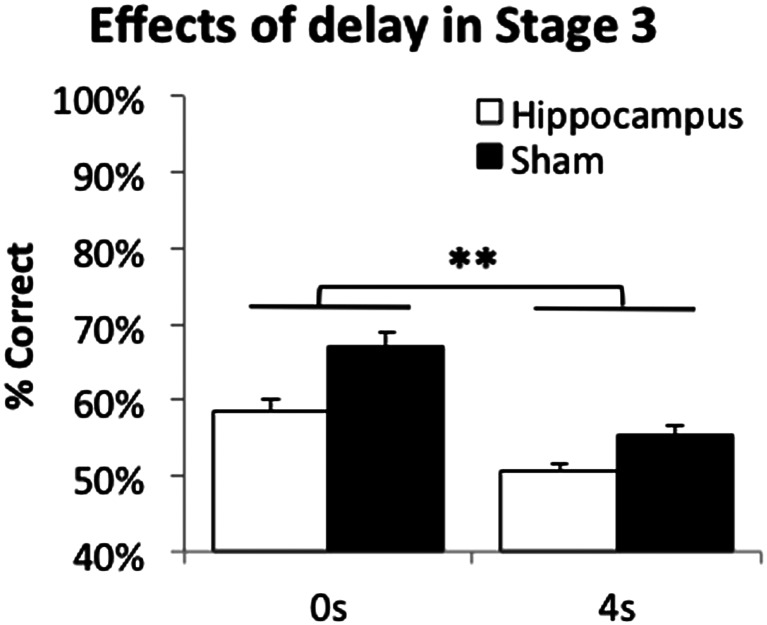
Effects of hippocampal lesion on TUNL (stage 3), when predictability of the correct location was low. Data are presented as mean ± standard error of the mean (SEM). **p < .005 main effect of lesion

One mouse from the hippocampal lesion group was excluded due to rectal prolapse. There were main effects of lesion and delay (*F*(1,27) = 17.71, *p* < .001; *F*(1,27) = 57.86, *p* < .001, respectively; see Fig. 6), but no lesion by delay interaction (*F*(1,27) = 2.10, *p* = .159). Thus, consistent with the findings from experiment 2, the TUNL task was highly sensitive to hippocampal lesions, with hippocampus-lesioned mice impaired even in the 0-s delay condition.

### Experiment 4: sample non-centre locations only (stage 1)

Following experiment 3, we studied the effects of delay and separation in highly predictable conditions, i.e. stage 1. One of the benefits of stage 1 is that it is relatively easy and quick to train mice on this compared to stages 2 and 3. Thus, if this method is sufficiently sensitive to hippocampal dysfunction, further training on higher stages may not be necessary in some situations.

**Fig. 7:**
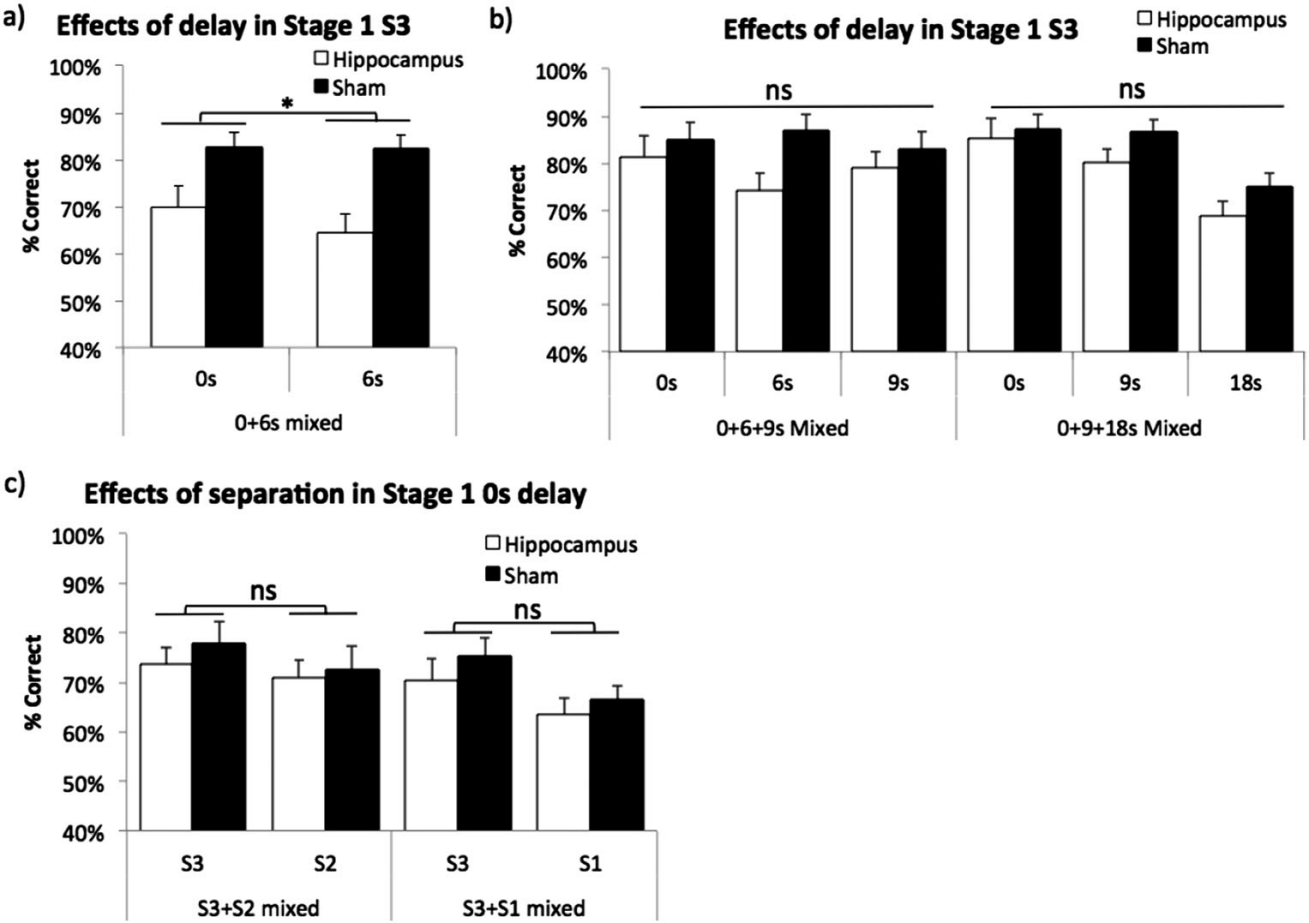
Effects of hippocampal lesion on TUNL (stage 1), when predictability of the correct location was high. **a** Initial mixed-delay sessions (0 and 6 s mixed); **b** after extended training in two mixed-delay conditions (0, 6, 9 s mixed; 0, 9, 18 s mixed); **c** two mixed spatial separation conditions (S3 and S2 mixed; S3 and S1 mixed). Data are presented as mean ± standard error of the mean (SEM). *ns* denotes not significant, *p < .05 main effect of lesion

Two mice from the sham group were excluded from testing due to rectal prolapse. First, mixed 0 and 6 s delay sessions revealed a main effect of lesion (*F*(1,25) = 9.25, *p* = .005), but there was neither a main effect of delay nor an interaction (*F*(1,25) = 2.84, *p* = .105; *F*(1,25) = 2.49, *p* = .127, respectively, see Fig. 7a). Second, in the mixed 0, 6, 9 s delay condition, there was no main effect of lesion or delay (*F*(1,25) = 1.87, *p* = .184; *F*(1.56,38.99) = 1.04, *p* = .346, respectively, see Fig. 7b). Lastly, with longer delays of 9 and 18 s, there was a main effect of delay (*F*(1.60,39.96) = 25.02, *p* < .001), but neither a main effect of lesion nor a lesion by delay interaction was found (*F*(1,25) = 1.68, *p* = .206; *F*(1.60,39.96) < 1, ns, respectively, see Fig. 7b).

Next, two mixed-separation level conditions were run with a fixed 0-s delay. In both mixed S3 and S2 and mixed S3 and S1 conditions, there were main effects of separation level (*F*(1,25) = 9.06, *p* = .006; *F*(1,25) = 12.58, *p* = .002, respectively, see Fig. 7c). But neither main effect of lesion nor lesion by separation level interactions were significant in the two conditions (*F*(1,25) < 1, ns; *F*(1,25) = 1.10, *p* = .304, respectively, in the S3 and S2 condition; *F*(1,25) < 1, ns; *F*(1,25) < 1, ns, respectively, in the S3 and S1 condition).

To summarise, some of the results of experiments 2 and 3 suggested that sample centre trials were more sensitive to hippocampal dysfunction than non-centre trials. This is perhaps not surprising as the correct location on sample centre trials is less “predictable” than on sample non-centre trials. However in this experiment (experiment 4), sessions of entirely sample non-centre trials also initially yielded strong effects of hippocampus lesions. But it should be noted that over training, the difference between lesion and sham groups became non-significant. It appears that mice with hippocampal dysfunction may be able to cope with relatively long delays in sample non-centre trials if sufficiently trained. This finding suggests that the optimal testing method should involve both sample centre and sample non-centre trials.

## Discussion

The main findings of the present study are that mice, like rats (Talpos et al. 2010), can be trained on the touchscreen TUNL task and eventually use multiple locations in a similar manner to rats (Talpos et al. 2010). In addition, the task was found to be highly sensitive to hippocampal dysfunction in mice. Indeed, it is so sensitive that it was difficult to find task conditions under which hippocampus-lesioned mice were not impaired. However, many manipulations that might be studied using this task—gene deletion, selective pharmacology, etc.—will likely induce much more subtle hippocampal dysfunction and in such cases high sensitivity may be an advantage.

The effects of dHp dysfunction on TUNL dissociated between sample centre and sample non-centre trials in some cases. For example, in the mixed-separation session of experiment 2, only sample centre trials were hippocampus-sensitive (see Fig. 4c, d). Experiments 3 and 4 then revealed that dHp lesioned mice were more consistently impaired in sample centre trials than in sample non-centre trials (although this may have been due at least in part to extended training in experiment 4). This pattern of results might be explained in terms of “predictability” of the choice location: when the sample location is in the centre, the correct location could be either to the right or to the left side of the sample location (low predictability), but when the sample location is off-centre, the correct location is more likely to be to the left if the sample is to the right of centre or to the right if the sample is to the left of centre (high predictability). Thus, presenting the sample away from centre may make the task easier, consistent with better percent correct scores overall on sample non-centre trials. Furthermore, it is possible that sample non-centre trials could be solved using more egocentric strategies (e.g. “leftwards” versus “rightwards”, rendering these trials less hippocampus-sensitive under some conditions).

Another possibility is that centre locations have fewer local cues with which to define and remember them, whereas sample locations presented away from centre, i.e. sample non-centre, are closer to the walls of the chamber. Therefore, the wall cues can be used more readily to remember the sample non-centre locations, rendering such trials less hippocampus-dependent in at least some situations. Whatever the reason, the results suggest that both sample centre and sample non-centre trials should be included in TUNL sessions, and that analysing these trial types separately might yield patterns of effects that might otherwise be missed if data from these trials are averaged together.

It remains unclear why, in our pilot studies (Romberg, unpublished), mice were unable to learn the task under the same conditions as the rat; indeed, it is our experience that mice perform very well compared to the rat on other touchscreen tasks (Bartko et al. 2011; Coba et al. 2012; Nithianantharajah et al. 2013; Romberg et al. 2013). One possibility is that the task as experienced by rat and mouse differ in spatial scale: the screen-to-body size ratio is greater for the mouse than the rat in touchscreen chamber systems (Horner et al. 2013). This could conceivably alter the task demands in a way that renders the task more difficult for the mouse. Although in the present study we had success using a different training method from that used with the rat, other solutions might be possible, for example involving altering the spatial scale so as to be more compatible with the size of a mouse. Alternatively, other task parameters we did not manipulate, such as increasing inter-trial interval to reduce interference, could have a positive effect.

### Pharmacological studies using TUNL

TUNL is well-suited to pharmacological studies as mice can be trained to a criterion and tested repeatedly on a stable performance baseline. Repeated exposures to a task frequently occur while testing multiple drugs or escalating multiple doses of a drug on the same animals. This may render development of mediating strategies (Chudasama and Muir 1997) over time that could confound behavioural effects of pharmacological investigations. However, the TUNL task in mice has detected the effects of hippocampal dysfunction throughout the whole duration of the present study. This was more consistent in the sample centre trials that might be resistant to mediating strategies due to low predictability of to-be-correct locations. In addition, task difficulty can be manipulated parametrically by varying parameters including delay and stimulus separation. This feature can be particularly useful for bringing performance down from ceiling to investigate improvements in performance by potential cognitive enhancers. How exactly a researcher designs and runs an experiment with any behavioural test, including TUNL, will of course depend on the question being asked, the animal model, the manipulations made and measurements taken, and other considerations. Detailed suggestions regarding the experimental design and step-by-step procedures for various kinds of experiments using TUNL were described in a recent protocol paper (Oomen et al. 2013). We have provided a brief example to test acute effects of cognitive enhancers on TUNL performance using a disease model with pre-existing pathology in the “Supplementary Materials”.

No pharmacological data are available yet for the mouse, but data from the rat are promising. For example, in a “continuous” version of TUNL (cTUNL; Oomen et al. this issue), temporary mPFC inactivation by the GABA agonists muscimol and baclofen resulted in a delay-dependent performance deficit (performance affected at long, but not short delays). Infusion of the α1 receptor agonist phenylephrine into prefrontal cortex significantly improved performance of the same task (Hvoslef-Eide et al. this issue). This was specific to trials where working memory was taxed using long delays, and the separation between stimuli was large. The effect of α1 agonism contrasted with that of the α2 receptor agonist guanfacine, which had little effect. In another study, rats administered with methylazoxymethanol acetate (MAM) on embryonic day 17, used as a pharmacological model of schizophrenia, were shown to be impaired at both acquisition and performance of cTUNL (Howe et al. this issue).

The present study showing that in mice, like rats (Talpos et al. 2010), TUNL is sensitive to hippocampus dysfunction suggests that TUNL requires similar neural circuitry in mice and rats. Therefore, it is highly likely that mouse TUNL will prove equally useful in pharmacological investigations.

### General considerations for using TUNL with mice

The findings of the present study provide the basis for a number of suggestions for researchers using TUNL with mice.

Based on our results, we suggest that mice are trained on TUNL using a method somewhat different from that used to train the rat (Talpos et al. 2010). Specifically, a restricted set of parameters, such as those in stage 1 or 2 in this paper, i.e. one row of five locations (see Fig. 1 and Table 1), is suggested to avoid the most difficult, smaller separation levels in the early training phases. Once mice acquire TUNL, further manipulations can be carried out by varying delay, spatial separation level and ITI (McAllister et al. 2013). At least one other group has reported success training mice on this task in a similar, simplified manner (Leach and Crawley 2014). Note that although we had success with one method for the mouse, it may be that by varying other task parameters, alternative or additional improvements could be made.

We found that trials in which the sample is in the centre of the display are more consistently sensitive to hippocampal dysfunction than sample non-centre trials (e.g. experiment 2, Fig. 4d). Therefore, researchers may wish, especially if hippocampal function is of interest, to pay particular attention to sample centre trials.

However, this is not to suggest that only sample centre trials should be used. Indeed, repeated use of sample centre-only trials would be expected to engender location biases on choice (i.e. away from centre) as there would be higher reward density in these locations. (Similarly, using sample non-centre trials only would be expected to lead to biased on choice toward the centre.) In general, within a session, each location should be balanced for the number of correct and incorrect locations in the choice phases.

Moreover, ideally the number of these two trial types should be equal across separation level. Without an appropriate control of trial numbers for each trial type, small separations tend to have more sample centre trials, which may confound the interpretation of spatial separation.

## Electronic supplementary material

ESM 1 (DOCX 119 kb)
