# Supplementary material for: Trial-unique, delayed nonmatching-to-location (TUNL) touchscreen testing for mice: sensitivity to dorsal hippocampal dysfunction
Source: Psychopharmacology (Berl). 2015 Jul 15;232(21-22):3935–45. doi: 10.1007/s00213-015-4017-8 (PMC4600470; doi:10.1007/s00213-015-4017-8)
Supplement: Supplementary file 1 — (DOCX 119 kb) [file 213_2015_4017_MOESM1_ESM.docx]

**Supplementary Material**

**A case scenario for a pharmacological study using mouse disease model**

The consistent testing environment and well-controlled automated nature of testing in touchscreen boxes is an advantage when running pharmacological studies. TUNL is highly hippocampus-sensitive and has already been shown to be sensitive to pharmacological manipulations, which can both impair and improve TUNL performance. Detailed suggestions regarding the experimental design and step-by-step procedures for various kinds of experiments using TUNL were described in a recent protocol paper (Oomen et al. 2013). How exactly a researcher designs and runs an experiment with any behavioural test, including TUNL, will of course depend on the question being asked, the animal model, the manipulations made and measurements taken, and other considerations. Here we provide a brief example to test acute effects of cognitive enhancers on TUNL performance using a disease model with pre-existing pathology.

1. Pretraining as described in the Methods section.

2. Progress toward a ‘baseline’ TUNL setting through successive training steps as was done in the current study, i.e., Stage 1 (S3 🡪 S2 🡪 S1) 🡪 Stage 2 (S1 🡪 S0) 🡪 Stage 3 (see Table 1). An ideal baseline TUNL setting would be Stage 2 or beyond including sample centre trials. Task performance between the disease and control groups should not differ statistically. From this anchoring point, delay and/or spatial separation can be manipulated to find any spatial working memory or pattern separation deficits in the disease model group. In addition, ideally both disease model and control groups should advance to the next step on the same day rather than animals progressing individually (thus avoiding a situation in which later pharmacological effects are potentially confounded by differences in behavioural training experience).

3. Ensure any deficits found in 2 are stable, e.g., steady over a minimum of 5-7 sessions, before starting pharmacological investigations.

4. Pharmacological manipulations using latin square or other controlled designs.
